# Mitochondrial genome sequencing of a vermivorous cone snail *Conus quercinus* supports the correlative analysis between phylogenetic relationships and dietary types of *Conus* species

**DOI:** 10.1371/journal.pone.0193053

**Published:** 2018-07-30

**Authors:** Bingmiao Gao, Chao Peng, Qin Chen, Junqing Zhang, Qiong Shi

**Affiliations:** 1 Hainan Provincial Key Laboratory of Research and Development of Tropical Medicinal Plants, Hainan Medical University, Haikou, China; 2 Shenzhen Key Lab of Marine Genomics, Guangdong Provincial Key Lab of Molecular Breeding in Marine Economic Animals, BGI Academy of Marine Sciences, BGI Marine, BGI, Shenzhen, China; 3 School of Agricultural and Forestry Science and Technology, Hainan Radio & TV University, Haikou, China; Agriculture and Agri-Food Canada, CANADA

## Abstract

Complete mitochondrial genome (mitogenome) sequence of a worm-hunting cone snail, *Conus quercinus*, was reported in this study. Its mitogenome, the longest one (16,460 bp) among reported *Conus* specie, is composed of 13 protein-coding genes (PCGs), 22 transfer RNA (tRNA) genes, two ribosomal RNA (rRNA) genes and one D-loop region. The mitochondrial gene arrangement is highly-conserved and identical to other reported. However, the D-loop region of *C*. *quercinus* is the longest (943 bp) with the higher A+T content (71.3%) and a long AT tandem repeat stretch (68 bp). Subsequent phylogenetic analysis demonstrated that three different dietary types (vermivorous, molluscivorous and piscivorous) of cone snails are clustered separately, suggesting that the phylogenetics of cone snails is related to their dietary types. In conclusion, our current work improves our understanding of the mitogenomic structure and evolutionary status of the vermivorous *C*. *quercinus*, which support the putative hypothesis that the *Conus* ancestor was vermivorous.

## Introduction

Cone snails (*Conus spp*.), a species-rich genus of venomous marine gastropods, produce a complex of conotoxins for prey capture and defense. They are usually classified into fish-hunting (piscivorous), snail-hunting (molluscivorous) and worm-hunting (vermivorous) groups [1–3]. The number of piscivorous species is the least, while these snails are assessed as deadly to humans. A larger number of molluscivorous species is dangerous, although some snails have been implicated in unconfirmed fatalities. Forming the largest group, the vermivorous species account for 70% of the *Conus* genus, while they seem to be nonthreatening [2–4]. There are more than 800 *Conus* species, and each typically contains 100~200 venom peptides; therefore, a total of over 80,000 conotoxins have been identified from various cone snails around the world [5,6].

The colors and diets, along with the composed conotoxins, in different cone snails are abundant and complicated; hence, related taxonomy, population genetics, evolutionary biology and phylogenetics have aroused the interest of scientists [7,8]. Evolutionary relationships between feeding and conotoxins have been discussed on basis of transcriptomics, proteomics and genomics [9,10]. The diversity of peptides in the venom of cone snails confirms that most species are able to produce a variety of conotoxins, as widely reported in literatures [11,12]. There is a specific hypothesis for the shift from an ancestral worm-hunting to more recent fish-hunting [13]. However, the poor performance of venom components in predicting prey taxonomic class suggests that conotoxins (gene superfamily) and traditional means of categorizing prey types (worms, mollusa, fish) do not accurately clarify the evolutionary dynamics between venom composition and diets [14–16].

Nowadays, mitochondrial genome (mitogenome) has been one of the most popular tools widely applied for gastropod mollusk taxonomy, population genetics, evolutionary biology and phylogenetics [17]. Gastropod mollusk mitogenomes usually exhibit high diversity of gene orders, and accordingly offer a suitable model system to study the patterns, rates, and mechanisms of mitogenome rearrangement as well as the phylogenetic utility of arrangement comparisons [18]. So far, the mitogenome sequences have been reported for nine cone snails, including two piscivorous (*C*. *consors* and *C*. *striatus*), three molluscivorous (*C*. *tulipa*, *C*. *textile* and *C*. *gloriamaris*), three vermivorous (*C*. *borgesi*, *C*. *capitaneus* and *C*. *tribblei*), and one broad dietary (*C*. *californicus*) species [19–26].

Here, we reported the mitogenome of an additional vermivorous cone snail, *C*. *quercinus*, and described some outstanding features of its mitogenome sequence. Related mitogenomic structure and phylogentic status are going to provide more supportive evidence for the putative hypothesis about the vermivorous *Conus* ancestor and the correlation between traditional classification of prey types and mitogenome evolution.

## Materials and methods

### Ethical statement

No specific permits were required for the collection of specimens for this study. These *Conus* specimens are common in China, and the field collection did not involve any endangered or protected species. Our experimental procedures complied with the current laws on animal welfare and research in China, and were specifically approved by the Animal Research Ethics Committee of Hainan Medical University.

### Genomic DNA extraction and sequencing

Live *C*. *quercinus* were collected in the offshore areas of Lingshui City, Hainan Province, China. Around 150 mg of foot tissue was ground to powder using mortar and pestle under liquid nitrogen. Total genomic DNA was extracted by the Column mtDNAout kit (Tianda, Beijing, China) according to the manufacturer’s instructions with minor modifications. The purified genomic DNA was quantified with a Nanodrop 2000 spectrometer (ThermoFisher Scientific, Wilmington, DE, USA).

Normalized DNA of 3 μg was employed to prepare a paired-end library using the NEB Next DNA sample libraries kit (New England Biolabs, New England) in accordance with the manufacturer’s instructions. Quantification and size estimation of the library were performed on a Bioanalyzer 2100 High Sensitivity DNA chip (Agilent, Palo Alto, CA, USA). Finally, the library was normalized to 2 nM and sequenced on the Illumina HiSeq2000 (Illumina, San Diego, CA, USA).

### Sequence assembly

Illumina paired-end reads were filtered on the basis of quality values, and the low-quality bases (quality < 20, *p*_error_> 0.01) at upstream and downstream were trimmed. The remained clean data were *de novo* assembled by SOAPdenovo2 (http://soap.genomics.org.cn/soapdenovo.html) on the basis of overlapping and paired-end relationships. All the cleaned reads were also mapped onto the assembled contigs with Bowtie 2 (2.2.5) [27] to estimate the sequencing depth. Those contigs with sequencing depth over 30× were mapped to the Conoidea mitochondrial genomes that were downloaded from the NCBI non-redundant nucleotide database (Nt) with blastn (2.2.31+) to validate mito-contigs, and the remaining genome gaps were filled with a python script.

Genome confirmation was indispensable to perform after assembling. Finally, the paired-end clean reads were mapped onto the assembled genome with 100% coverage, and the insert-size matched the information of sequenced library. The sequencing depth, coverage and relationship of the paired-end reads were the main criteria for confirmation.

### Genome annotation and analysis

Preliminary gene annotation was realized through the online program Dual Organellar GenoMe Annotator (DOGMA) [28] and ORF Finder [29] with invertebrate mitochondrial genetic codes and default parameters. To verify the exact gene and exon boundaries, putative nucleotide and protein sequences were BLAST searched in the public Nt and Nr (the NCBI Non-redundant protein) databases. All tRNA genes were further confirmed through online tRNAscan-SE and ARWEN search server [30–32], in combination with the annotated results of ARAGORN. Graphical map of the circular plastome was drawn with Organellar Genome DRAW (OGDRAW v1.2) [33]. Relative synonymous codon usage (RSCU) value was employed to evaluate the synonymous codon bias in accordance with a previous report [34]. The skewing of nucleotide composition was calculated according to the following formulas: AT skew = (A–T)/(A+T) and GC skew = (G−C)/(G+C) [35, 36]. To further analyze the evolutionary adaptation in the *Conus* lineage, we applied DnaSP 6 [37] to estimate the ratios of non-synonymous (Ka) and synonymous (Ks) substitutions in the mitochondrial genomes among cone snails with the three different dietary types.

### Phylogenetic analysis

Phylogenetic analysis was performed among ten taxa in the Conidae based on the nucleotide sequences of eleven protein-coding genes without ATP8 and ND6 from GenBank, and *Oxymeris dimidiata* (NC_013239.1), *Fusiturris similis* (NC_013242.1) and *Lophiotoma cerithiformis* (NC_008098.1) were employed as the out-groups. Before reconstructing phylogenetic trees, both nucleotide and protein sequences of eleven protein-coding genes were subjected to concatenated alignments using MUSCLE 3.8.31 (http://www.drive5.com/muscle/) [38]. The best-fit model GTR+G+I for nucleotide sequences was selected using the Akaike Information Criterion (AIC) with jModeltest [39]. Bayesian analyses of both nucleotide and protein alignments were carried out using PhyloBayes version 3.3f [40] under the best-fit model. Two independent Markov Chain Monte Carlo (MCMC) chains were run simultaneously to determine whether the searching reached stabilization, and were immediately stopped when all chains converged (maxdiff less than 0.1). The phylogenetic trees were eventually constructed using the Tree View program v.1.65 and Evolview (www.evolgenius.info/evolview/) [41].

## Results and discussion

### Genome organization and nucleotide composition

**Fig 1:**
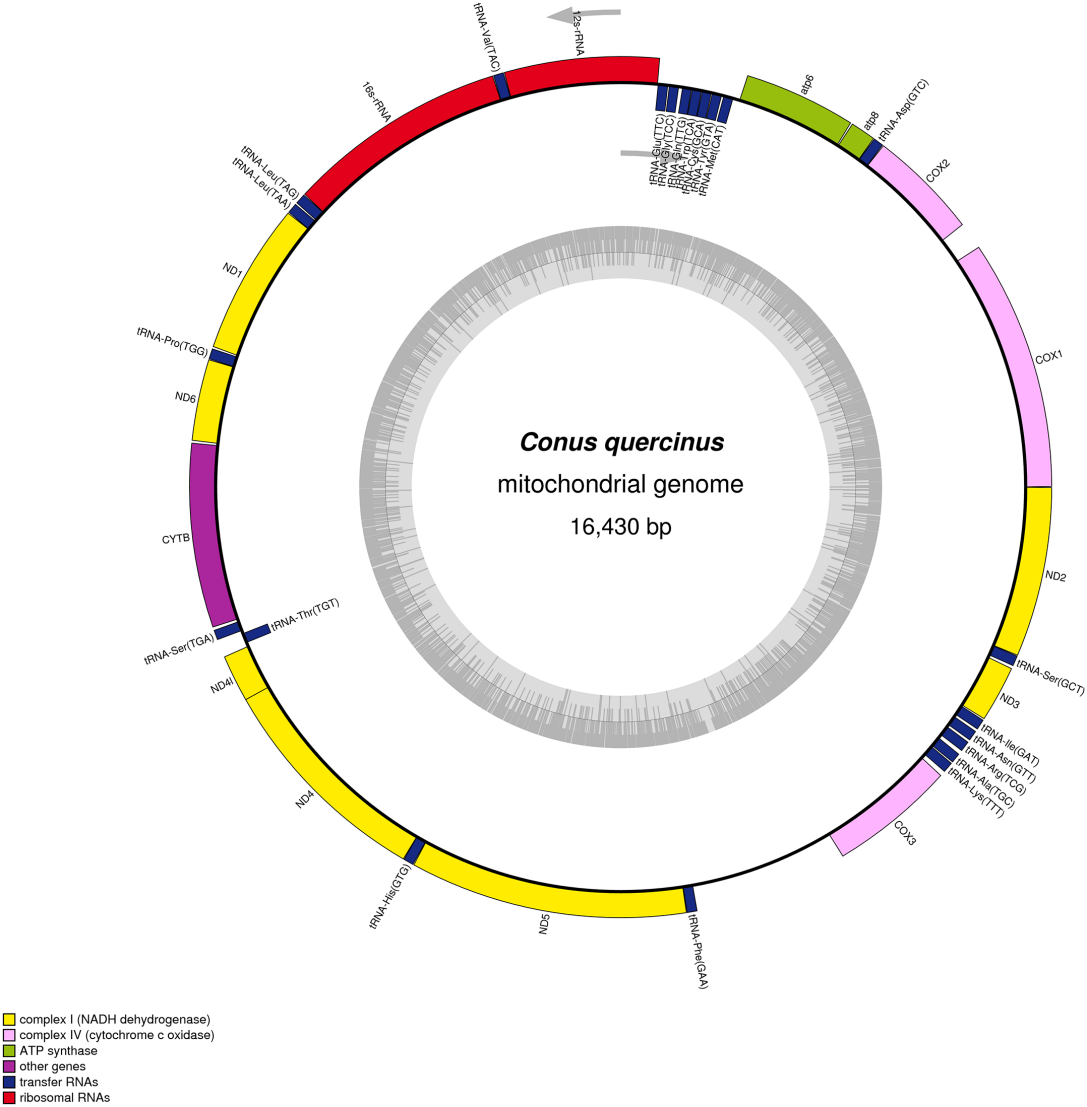
Mitochondrial map of *Conus quercinus*. Genes outside the map are transcribed in a clockwise direction, whereas those inside the map are transcribed counterclockwise. Gene blocks are filled with different colors as the cut line shows.

The mitogenome of *C*. *quercinus* is a closed circular molecule of 16,430 bp in length (GenBank accession No. KY609509; see more details in Fig 1). It encodes a high mutation region (D-loop), and a typical set of 37 mitochondrial genes including 13 protein-coding genes (PCGs), two ribosomal RNA (rRNA) genes (12S rRNA and 16S rRNA), and 22 transfer RNA (tRNA) genes. Eight tRNA genes are encoded on the light (L) strand, whereas the other genes are located on the heavy (H) strand (Table 1).

**Table 1:** The detailed mitogenome structure of *Conus quercinus*.

NO.	Gene	Strand	Position	Size (bp)	GC(%)	Amino Acids	Initiation Codon	Termination Codon	Anti-codon	Intergenic nucleotide (bp)*
1	COX1	H	1–1548	1548	37.14%	515	ATG	TAG		162
2	COX2	H	1711–2397	687	34.93%	228	ATG	TAA		0
3	tRNA-Asp	H	2398–2464	67	28.36%				GTC	0
4	atp8	H	2465–2626	162	25.31%	53	ATG	TAA		6
5	atp6	H	2633–3328	696	32.90%	231	ATG	TAA		41
6	tRNA-Met	L	3370–3436	67	26.87%				CAT	9
7	tRNA-Tyr	L	3446–3512	67	40.30%				GTA	1
8	tRNA-Cys	L	3514–3578	65	30.77%				GCA	0
9	tRNA-Trp	L	3579–3644	66	33.33%				TCA	0
10	tRNA-Gln	L	3645–3708	64	40.63%				TTG	20
11	tRNA-Gly	L	3729–3794	66	27.27%				TCC	5
12	tRNA-Glu	L	3800–3867	68	35.29%				TTC	0
13	12s-rRNA	H	3868–4825	958	35.18%					0
14	tRNA-Val	H	4826–4892	67	16.42%				TAC	0
15	16s-rRNA	H	4893–6262	1370	30.73%					0
16	tRNA-Leu	H	6263–6332	70	30.00%				TAG	6
17	tRNA-Leu	H	6339–6407	69	31.88%				TAA	0
18	ND1	H	6408–7349	942	33.01%	313	ATG	TAG		8
19	tRNA-Pro	H	7358–7426	69	40.58%				TGG	0
20	ND6	H	7427–7933	507	28.01%	168	ATG	TAA		11
21	CYTB	H	7945–9084	1140	35.00%	379	ATG	TAA		15
22	tRNA-Ser	H	9100–9164	65	49.23%				TGA	16
23	tRNA-Thr	L	9181–9248	68	38.24%				TGT	23
24	ND4l	H	9272–9568	297	31.31%	98	ATG	TAG		-7
25	ND4	H	9562–10944	1383	32.39%	460	ATG	TAG		-1
26	tRNA-His	H	10944–11010	67	31.34%				GTG	0
27	ND5	H	11011–12726	1716	33.39%	571	ATG	TAA		-1
28	tRNA-Phe	H	12726–12791	66	36.36%				GAA	0
29	D-loop	H	12792–13734	943	28.74%					0
30	COX3	H	13735–14514	780	41.67%	259	ATG	TAA		0
31	tRNA-Lys	H	14542–14611	70	37.14%				TTT	27
32	tRNA-Ala	H	14617–14683	67	34.33%				TGC	5
33	tRNA-Arg	H	14706–14774	69	44.93%				TCG	22
34	tRNA-Asn	H	14787–14854	68	36.76%				GTT	12
35	tRNA-Ile	H	14868–14936	69	43.48%				GAT	13
36	ND3	H	14942–15295	354	34.75%	117	ATG	TAG		5
37	tRNA-Ser	H	15310–15377	68	44.12%				GCT	14
38	ND2	H	15378–16430	1053	34.38%	350	ATG	TAA		0

*Note: The positive intergenic nucleotide value means the number of bases until the next gene, while the negative value represents the number of bases overlapped between two genes.

In the *C*. *quercinus* mitogenome, gene overlapping occurred three times (the negative numbers in Table 1), spanning 1~7 nucleotides (nts), for a total of 9 nts. The intergenic spacer region occurred 20 times (the positive numbers in Table 1), spanning 1~162 bp, for a total of 421 bp. The overall base composition is estimated to be 28.15% for A, 38.32% for T, 14.82% for C and 18.71% for G, with a high A+T content at 66.47% (Table 2).

**Table 2:** Nucleotide composition of *Conus* mitogenomes.

Species	Accession No.	Length(bp)	A(%)	T(%)	C(%)	G(%)	AT(%)	AT-skew	GC-skew
*C*. *consors* (P)	NC_023460.1	16,112	27.72	39.39	13.52	19.36	67.11	-0.1738	0.1774
*C*. *striatus* (P)	NC_030536.1	15,738	25.93	38.61	14.64	20.82	64.54	-0.1964	0.1743
*C*. *tulipa* (P)	NC_027518.2	15,756	28.55	37.85	15.11	18.49	66.40	-0.1401	0.1005
*C*. *gloriamaris* (M)	NC_030213.1	15,774	27.73	38.54	15.04	18.70	66.27	-0.1631	0.1084
*C*. *textile*(M)	NC_008797.1	15,562	27.24	37.95	15.65	19.16	65.19	-0.1643	0.1006
*C*. *tribblei* (V)	NC_027957.1	15,570	28.10	37.85	15.32	18.73	65.95	-0.1478	0.1000
*C*. *borgesi* (V)	NC_013243.1	15,536	28.66	38.49	14.60	18.24	67.15	-0.1464	0.1107
*C*. *capitaneus* (V)	NC_030354.1	15,829	25.60	36.62	16.29	21.49	62.22	-0.1771	0.1376
***C*. *quercinus* (V)**	**KY609509**	**16,430**	**28.15**	**38.32**	**14.82**	**18.71**	**66.47**	**-0.1530**	**0.1160**
*C*. *californicus*(B)	NC_032377.1	15,444	28.59	37.19	16.76	17.46	65.78	-0.1307	0.0205

Note: M, molluscivorous; P, piscivorous; V, vermivorous. B, broad with a combination of molluscivorous, piscivorous and vermivorous types.

Complete mitogenomes of the ten *Conus* species, including *C*. *quercinus* and nine previously reported [19–26], displayed moderate size variation, with the mean size of 15,755 bp (SD = 298.4, n = 10), ranging from 15,444 bp (*C*. *californicus*) to 16,430 bp (*C*. *quercinus*). AT and GC skews are measures of compositional asymmetry. In the *C*. *quercinus* mitogenome, GC-skew values are always positive, while the values of AT-skew are negative (Table 2).

The mitogenome gene arrangement is conserved and identical to those of other reported *Conus* species. The intergenic sequences vary between 0 and 41 nts, and one relatively large region of 162 nts happens between COX1 and COX2 (Fig 1). The gene sequences of NADH dehydrogenase subunit 4L (NAD4l) and subunit 4 (NAD4) are overlapped by 7 nts, NAD4 and tRNA-His by 1 nt, and NAD5 and tRNA-Phe by 1 nt.

### Protein-coding, tRNA and rRNA genes

**Fig 2:**
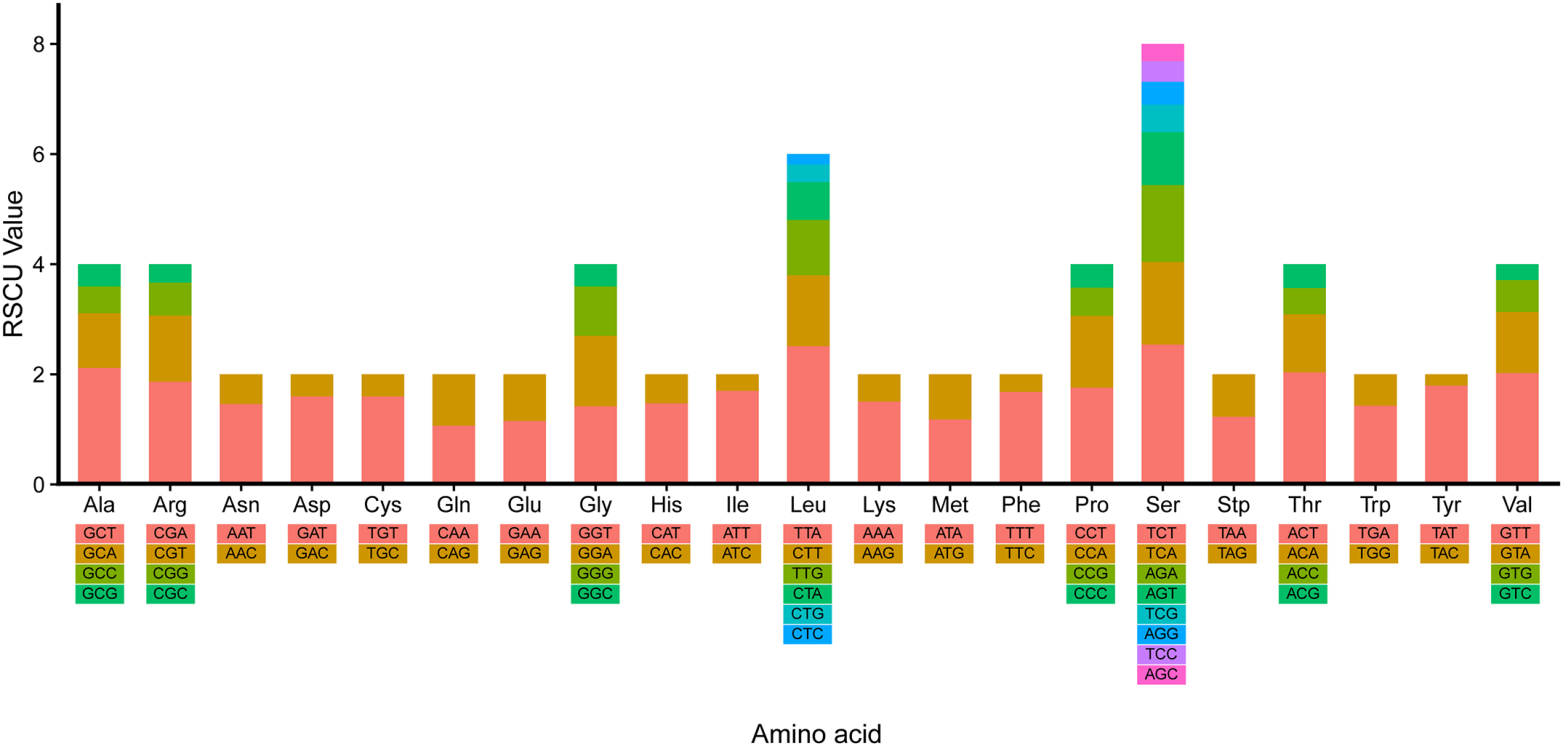
RSCU values in the mitogenome of *Conus quercinus*. Codon families are indicated below the X-axis.

The 13 PCGs of *C*. *quercinus* are similar in length and arrangement to the nine previously sequenced *Conus* mitogenomes. All PCGs are transcribed from the H strand in *C*. *quercinus*, with initiation of the standard start codon ATG. They also display the typical TAN termination codon, in which 8 PCGs have the complete termination codon TAA and 5 PCGs have the TAG (Fig 1 and Table 1). The RSCU values of the *C*. *quercinus* mitogenome were calculated (Fig 2), indicating that TTA (Leu), TCT (Ser), GCT (Ala), ACT (Thr), and GTT (Val) are the five most frequently used codons.

### D-loop region of the *C*. *quercinus* mitogenome

**Fig 3:**
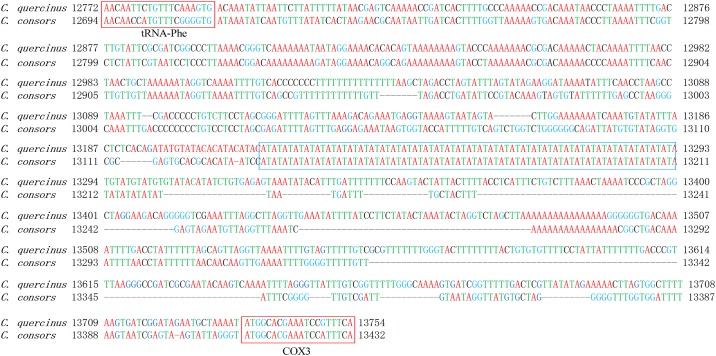
Scheme of the D-loop region in *C*. *quercinus* compared with *C*. *consors*. The region spans 943 bp and exhibits several outstanding motifs. The upper and lower red boxes denote the tRNA-Phe and COX3, and the blue box points to a long AT tandem repeat stretch.

The D-loop region between tRNA-Phe and COX3 in *C*. *quercinus* (Fig 1) is the longest (943 bp), which is much higher than those in other *Conus* species (97~698 bp; see more details in Table 3). Based on the sequence alignment of *C*. *quercinus* with other *Conus* species, we observed that the intergenic sequences of the *C*. *quercinus* mitogenome are also the longest in these *Conus* species. Usually, in animal mitochondrial genomes, the longest intergenic sequences were reported to play a key role in the initiation of replication and transcription [22, 42]. Interestingly, the D-loop region in *C*. *quercinus* (among the 10 *Conus* species except *C*. *californicus*) also presents the higher A+T content (71.3%) with a long AT tandem repeat stretch (68 bp; Fig 3).

**Table 3:** D-loop length of *Conus* mitogenomes.

Species	Start	End	Length (bp)	A+T (%)
***C*. *quercinus***	**12,792**	**13,734**	**943**	**71.3**
*C*. *consors*	12,714	13,412	698	70.3
*C*. *capitaneus*	12,801	13,142	342	63.7
*C*. *gloriamaris*	12,750	13,084	335	65.9
*C*. *striatus*	12,715	13,047	333	60.9
*C*. *tulipa*	12,742	13,074	333	68.1
*C*. *tribblei*	12,723	12,891	169	68.6
*C*. *borgesi*	12,721	12,847	127	66.1
*C*. *textile*	12,760	12,885	126	67.4
*C*. *californicus*	12,695	12,791	97	77.3

### Synonymous and nonsynonymous substitutions

**Fig 4:**
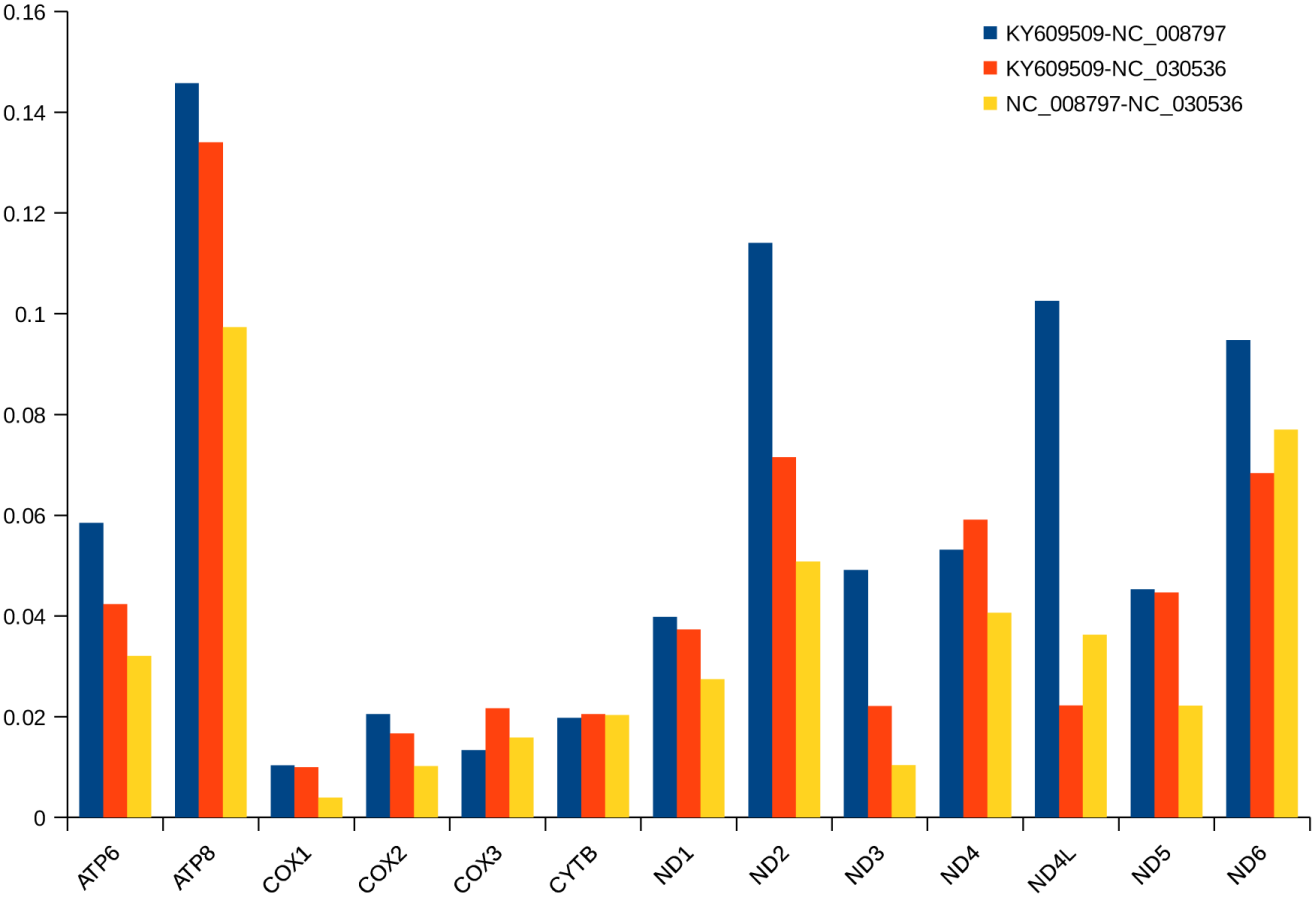
Ka/Ks ratios for the 13 mitochondrial PCGs among three representative *Conus* species. GenBank accession numbers: KY609509 for *C*. *quercinus*, NC_008797 for *C*. *textile*, and NC_030536 for *C*. *striatus*.

In genetics, the Ka/Ks ratio is of significance to estimate the balance between neutral mutations and is especially useful for understanding the evolutionary relations between homologous PCGs in closely related species [43]. To detect the influence of selection on the *C*. *quercinus*, Ka and Ks were estimated [44]. In all the 13 PCGs of three cone snails (Fig 4), the ratio of Ka/Ks is much less than 1 (between 0 and 0.16), indicating existence of a strong purifying or stabilizing selection.

**Fig 5:**
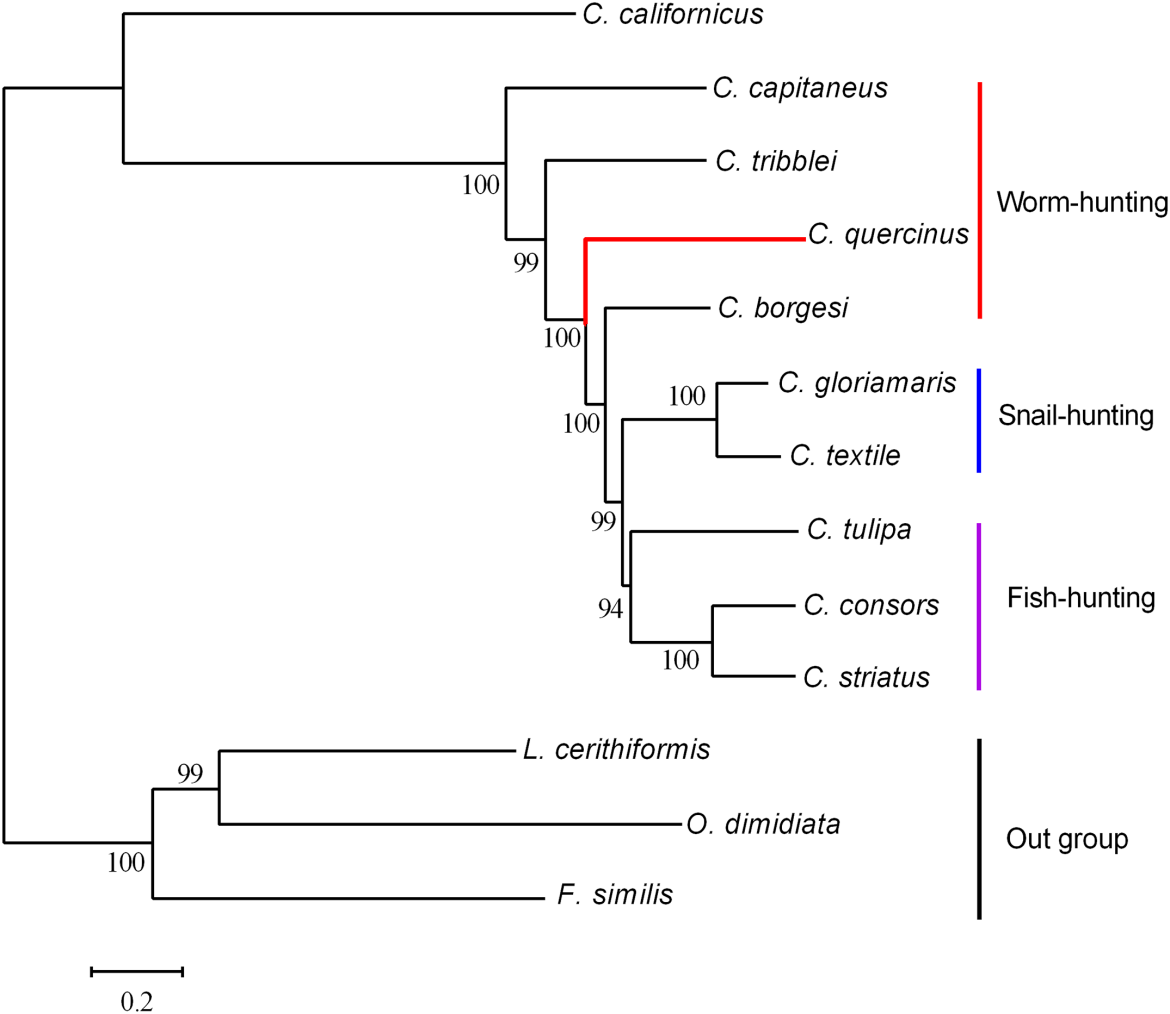
The phylogenetic tree of *Conus* species based on 13 complete mitogenome sequences. GenBank accession numbers: NC_008098.1 for *L*. *cerithiformis*, NC_008797.1 for *C*. *textile*, NC_013239.1 for *O*. *dimidiate*, NC_013242.1 for *F*. *similis*, NC_013243.1 for *C*. *borgesi*, NC_023460.1 for *C*. *consors*, NC_027518.2 for *C*. *tulipa*, NC_027957.1 for *C*. *tribblei*, NC_030213.1 for *C*. *gloriamaris*, NC_030354.1 for *C*. *capitaneus*, NC_030536.1 for *C*. *striatus*, and NC_032377.1 for *C*. *californicus*. The red branch highlights our present study of *C*. *quercinus*. Please note that the outgroups *L*. *cerithiformis*, *O*. *dimidiata* and *F*. *similis* are allochthonous species. Branch lengths and topologies were obtained using the Bayesian inference analysis.

The average Ka/Ks in ATP8 is the highest among the 13 PCGs, suggesting that this protein is under the least selective pressure among all the mitochondrial genes. Interestingly, in *C*. *textile* and *C*. *striatus*, the ratio of Ka/Ks is the least in nine protein-coding genes (except COX3, CYTB, ND4L and ND6) compared to *C*. *quercinus*, indicating that these two cone snails have a closer phylogenetic relationship than *C*. *quercinus*. These data are consistent with their dietary difference (Fig 5).

### Phylogenetic relationships of *Conus* species with different dietary types

Molecular phylogeny of the taxonomy is a hypothesis of its evolutionary patterns and processes. The molecular-based phylogenetic tree can estimate divergence times and ancestral distributions, and provides evidence relevant to taxonomic hypotheses [8]. To further validate the mitogenome sequence of *C*. *quercinus* and understand the evolutionary history of the *Conus* species with different feeding ecologies, we constructed a phylogenetic tree using Bayesian inference analysis with 13 complete mitogenomes downloaded from the NCBI (Fig 5). It is obvious that three different dietary types (vermivorous, molluscivorous and piscivorous) of cone snails, except the broad *C*. *californicus*, are clustered separately. This is consistent with previous reports [43, 45] and supports the putative hypothesis that the cone snail ancestor was vermivorous. However, the inclusion of *C*. *californicus* in the phylogenetic tree analysis may bias the results, because it is often regarded as an atypical member of Conidae due to its extremely broad diet and distant phylogenetic relationship to the rest of Conidae [14, 26].

Venom composition across *Conus* species has been hypothesized to be shaped by prey type and dietary breadth. Several studies [14] proved a significant positive relationship between dietary breadth and measures of conotoxin complexity by transcriptome sequencing of venom duct from 12 *Conus* species. However, given the high evolutionary lability of venom toxins, it is unclear that a clear relationship between dietary preference and venom composition should be expected. The prey taxonomic class of *Conus* can be predicted by venom components, but the performance of prey taxonomic class in predicting venom components was poor [14–17, 26]. By far, we are certain that the selective pressures driven by diets play a major role in shaping evolutionary patterns in venom across the cone snails.

## Conclusion

In this present study, we sequenced and annotated the complete mitogenome of *C*. *quercinus*. We used other nine publically available *Conus* mitogenomes to illustrate the structure of *C*. *quercinus* mitogenome and investigated the evolutionary relationships among *Conus* species. Interestingly, the mitochondrial gene arrangement of *C*. *quercinus* is highly conserved and identical to other *Conus* species. However, the D-loop region (943 bp) of *C*. *quercinus* is the longest with the higher A+T content (71.3%) and a long AT tandem repeat stretch (68 bp). The phylogenetic tree (Fig 5) revealed that three different dietary types of cone snails are clustered separately, suggesting that the phylogenetics of cone snails is related to their dietary types. Our current work improves our understanding of the mitogenomic structure and evolutionary status of the vermivorous *C*. *quercinus*, which support the putative hypothesis that the *Conus* ancestor was vermivorous.

## Abbreviations

H: heavy
L: light
M: molluscivorous
Mitogenome: mitochondrial genome
nts: nucleotides
P: piscivorous
PCGs: protein-coding genes
rRNA: ribosomal RNA
RSCU: relative synonymous codon usage
tRNA: transfer RNA
V: vermivorous
